# Measuring Progress of Regulatory Convergence and Cooperation Among Asia–Pacific Economic Cooperation (APEC) Member Economies in the Context of the COVID-19 Pandemic

**DOI:** 10.1007/s43441-021-00285-w

**Published:** 2021-04-11

**Authors:** Sannie Siaw Foong Chong, Mirinea Kim, Michelle Limoli, Eric Obscherning, Patricia Wu, Lila Feisee, Nobumasa Nakashima, John C. W. Lim

**Affiliations:** 1 Asia Pacific Technical Regulatory Policy, Pharma Technical Regulatory Policy and International Operations, Roche Singapore Technical Operations, F. Hoffmann La-Roche Ltd, Singapore, Singapore; 2 National Institute of Food and Drug Safety Evaluation, Ministry of Food and Drug Safety, Cheongju-si, Republic of Korea; 3 U.S. Food and Drug Administration, Washington, DC USA; 4 APEC Life Sciences Innovation Forum, Washington, DC USA; 5 Biotechnology Innovation Organization, Washington, DC USA; 6 Pharmaceuticals and Medical Devices Agency, Tokyo, Japan; 7 Duke-NUS Medical School, Centre of Regulatory Excellence, Singapore, Singapore; 8 SingHealth Duke-NUS Global Health Institute, Singapore, Singapore; 9 Consortium for Clinical Research & Innovation Singapore, Singapore, Singapore

**Keywords:** APEC, Regulatory convergence, Regulatory cooperation, Key performance indicators, COVID-19

## Abstract

**Purpose:**

Regulatory convergence and cooperation among medical product regulatory authorities are essential to delivering safe and efficacious products quickly to patients. The COVID-19 pandemic highlights the urgent need for streamlined regulatory approval processes—which can be achieved in part through regulatory convergence and cooperation—both to accelerate availability of COVID-19 vaccines, treatments and diagnostics and to maintain the availability of the existing medical products unrelated to COVID-19.

**Methods:**

The Asia–Pacific Economic Cooperation (APEC) Life Sciences Innovation Forum (LSIF) established the Regulatory Harmonization Steering Committee (RHSC) in 2008 to advance regulatory convergence among APEC’s 21 member economies. Key performance indicators (KPIs) were developed in 2018 to measure convergence.

**Results:**

This paper reports survey results collected from KPI tracking in March 2020 from medical product regulatory authorities in all 21 APEC economies concerning areas of regulatory practice in which they could converge and cooperate. For example, from 2008 to 2020, there was a 14.3% increase in the number of APEC member economy regulatory authorities sharing Good Manufacturing Practices (GMP) Certificates and a 28% increase in the number of regulatory authorities accepting multisite licenses in that same period. In addition, this paper explores how APEC economies could realize a maximum level of regulatory convergence and cooperation.

**Conclusions:**

Convergence efforts within APEC can accelerate availability of medical products including that related to COVID-19 vaccines, treatments and diagnostics, while maintaining the availability of the existing medical products unrelated to COVID-19 vaccines and treatment. New KPIs and capability building are to be considered to enable a new era of innovation ushered in by COVID-19.

## Background

The Asia–Pacific Economic Cooperation (APEC) Life Sciences Innovation Forum (LSIF) established the Regulatory Harmonization Steering Committee (RHSC) in 2008 to advance regulatory convergence among APEC’s 21 member economies^1^. “Regulatory convergence” is defined by the RHSC as “a voluntary process whereby the regulatory requirements across economies become more aligned over time as authorities adopt internationally recognized technical guidance, standards and scientific principles and common or similar practices and procedures” [9]. In February 2018, RHSC representatives from medical product regulatory authorities (RAs) and the biopharmaceutical industry proposed a number of key performance indicators (KPIs) to measure the progress of regulatory convergence in the APEC region across four areas of best practice [8, 12]:

1. The removal of the Certificate of Pharmaceutical Product (CPP) when CPP is no longer used to replace full or partial review of importing economies to grant marketing authorization;
2. The use of the Good Manufacturing Practices (GMP) certificate issued by the Pharmaceutical Inspection Cooperation Scheme (PIC/S) network to reduce inspection burden among RAs and industry;
3. The management of multiple manufacturing sites under a single license from the RA; and,
4. The use of risk-based evaluation based on the reliance practices.

In January 2019 and March 2020, the APEC Harmonization Center (AHC)^2^ conducted a survey to measure these KPIs among RAs in all 21 APEC member economies [5]. The AHC also requested retrospective baseline data from 2008. The COVID-19 pandemic in 2020 has brought new public and political attention to the importance of regulatory convergence and cooperation, both of which can help streamline market approval processes and accelerate availability of medical products for COVID-19. In June 2020, the APEC LSIF issued a statement on COVID-19 [6], which noted the following with regard to its regulatory convergence efforts in the RHSC:

“In response to the pandemic, the LSIF is accelerating efforts to advance regulatory convergence. […] Regulatory convergence helps APEC economies avoid unnecessary duplication of regulatory reviews thereby allowing products to be approved more quickly. […] Regulatory convergence can also save precious public resources by tapping into the expertise and work of other high-performing regulators around the region. […] The LSIF reaffirms its commitment to advancing the convergence of the regulatory review and approval processes for safe and effective medical products, especially those essential to the COVID-19 response.”

## Methodology

At its February 2018 meeting in Singapore the APEC RHSC agreed to a set of Key Performance Indicators (KPI). These KPIs were developed by a task force composed of regulators, industry members, the LSIF Advisors and the AHC. At its August 2018 meeting in Brisbane, Australia the APEC RHSC agreed to an assessment framework on “APEC RHSC Progression Towards Regulatory Convergence”. This framework included a quantitative assessment consisting of the KPIs which RHSC agreed would be in the form of a survey instrument to measure progress towards convergence from 2008 (prior to RHSC activities) through 2019. The APEC RHSC charged the AHC to develop, circulate and compile the results of this survey instrument entitled *Survey on APEC Regulatory Harmonization Key Performance Indicator* with input from the task force. This survey has since been conducted each year thereafter.

## Survey Participants

The survey participants comprised the medical product RAs in all 21 APEC member economies.

### Materials

The survey instrument entitled *Survey on APEC Regulatory Harmonization Key Performance Indicator.* can be found at this link.^3^ The survey instrument consists of two sections with the first section designed to gather general information about the respondent. The second section consists of a questionnaire with 8 questions designed to determine (a) participation of respondents in the existing international harmonization forums; (b) the level of cooperation and collaboration between respondent regulatory authority and other regulatory authorities; and (c) the level of reliance practiced by the respondent regulatory authority.

### Procedure:

#### Developing the KPIs

In a paper entitled *Developing key performance indicators to measure the progress of regional regulatory convergence and cooperation in Asia Pacific Economic Co-operations (APEC)* [12],^4^ Chong et al., presented a detailed account of how the APEC RHSC developed the KPIs included in the survey instrument. The RHSC drew from the existing harmonization efforts, such as the International Council for Harmonisation^5^ (ICH) experience in regulatory convergence and the Pharmaceutical Inspection Co-operation Scheme^6^ (PIC/S) experience and also took into consideration best practices and feasible processes for convergence to develop its KPIs. In general, the paper identified four areas of appropriate regulatory practice in which APEC economies could converge. The four areas are related to the use of (1) Certificate of Pharmaceutical Product (CPP); (2) PIC/S membership; (3) managing multiple sites in one license; and (4) risk-based reliance evaluation system. These four areas were explored together with feasible processes to develop KPIs to measure the progress of convergence.

Questions 4 through 7 and 8–2 in the questionnaire aim to address the first three areas of regulatory practice indicated above.

As per Chong et al., the reliance of one regulatory authority on the assessment of another regulatory authority is a clear indicator of regulatory convergence. The use of assessment reports from other trusted regulatory authorities in all or some parts of the drug approval process reduces approval time and reduces time to market. Accordingly, questions 8-1 and 8-1-1 of the Survey measure this KPI. The questions are meant to determine first whether there is only a single pathway for approval in the APEC economy or multiple pathways; and second to further clarify whether those with multiple pathways employ reliance on parts or all of the assessment of another regulatory authority to make their determination.

International forums, such as the ICH, PIC/S and International Pharmaceutical Regulator Programme all focus on harmonizing various aspects of the drug approval process. Membership in these organizations among others would serve as a significant impetus towards convergence. Accordingly, questions 1–3 in the Survey measure membership in these organizations. Increased membership in these and other related harmonization organizations are considered to be an important driver of convergence amongst the APEC economies.

In general, the number of “Yes” answers indicate progress towards convergence, with the exception of the question related to the number of APEC economies using CPPs. In this case, a “No” answer shows progress towards convergence. The CPP was originally implemented by the World Health Organization (WHO) to accelerate the availability of new drugs in developing countries by providing evidence of quality to accelerate product to market. However, there are significant differences among APEC economies in regard to CPP timing, terminology and format which actually do the opposite. Therefore, the task force determined that moving away from CPP requirements indicates movement towards convergence.

#### Collecting the Data

The survey instrument entitled *Survey on APEC Regulatory Harmonization Key Performance Indicator* was circulated on January 17, 2020. Data was collected for 2008 (pre-RHSC activity) and Q1 2019 (after 10 years of RHSC activity) and updated with 2020 Q1 data. Responses were received from all APEC member economies. The respondents are listed below.

1. Therapeutic Goods Administration (TGA), Australia
2. Ministry of Health, Brunei Darussalam
3. Public Health Agency of Canada
4. Department of Science and Technology and International Cooperation, National Medical Product Administration (NMPA), People’s Republic of China
5. Institute of Public Health (ISP), Chile
6. Taiwan Food and Drug Administration, Ministry of Health and Welfare, Chinese Taipei
7. Department of Health, Hong Kong Special Administrative Region
8. Indonesia Food and Drug Authority, Republic of Indonesia
9. Ministry of Health, Labour and Welfare, Japan
10. National Institute of Food and Drug Safety Evaluation, Ministry of Food and Drug Safety, Republic of Korea
11. The Federal Committee for Protection of from Sanitary Risks (COFEPRIS), Mexico
12. Medsafe, Ministry of Health, New Zealand
13. Pharmaceutical Services Branch, National Department of Health, Papua New Guinea
14. Directorate General of Drug Supplies and Drugs (DIGEMID), Peru
15. Food and Drug Administration, Department of Health, Philippines
16. Division for the State Quality Control of Medical Products, Federal Service for Surveillance in Healthcare (Roszdravnadzor), Russian Federation
17. Health Products Regulation Group, Health Sciences Authority (HSA), Singapore
18. National Pharmaceutical Regulatory Agency (NPRA), Malaysia
19. Food and Drug Administration Thailand
20. U.S. Food and Drug Administration, USA
21. Drug Administration of Vietnam

## Results

The results reported and collated herewith are based on the responses provided by the official representatives of the member economies’ RAs.

**Fig.1:**
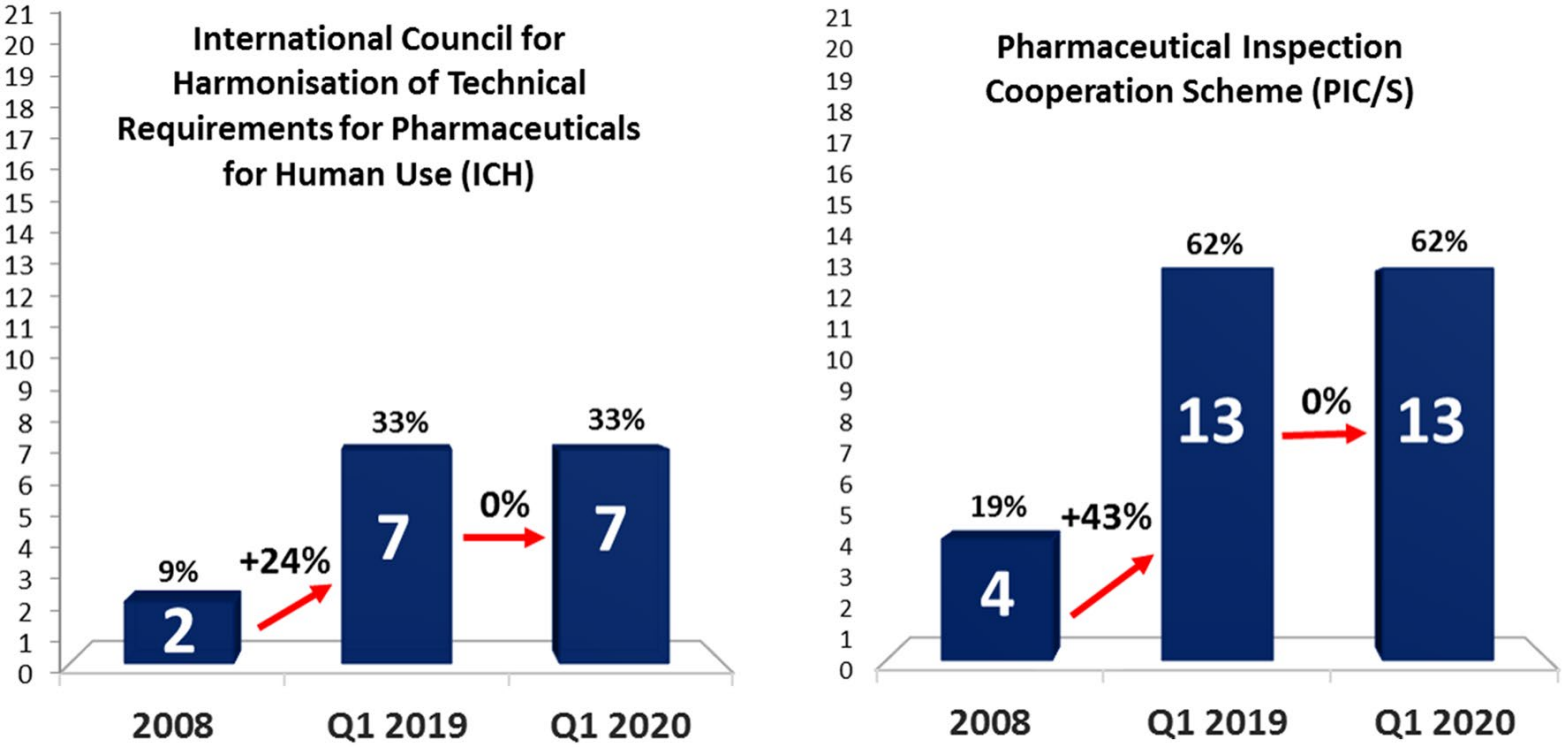
Increase in ICH and PIC/S membership among the APEC economies from 2008 to 2020

Membership of RAs in ICH and PIC/S builds collective confidence and trust, and enhances the necessary capacity to work toward regulatory convergence [16, 25]. In 2008, 9% of APEC RAs (2 of 21) were members of the ICH, while 19% (4 of 21) were members of PIC/S. Over the past decade the number of APEC RAs that are members of ICH and PIC/s have each more than tripled. To be exact, the increase in ICH and PIC/S membership from 2008 is 24% and 43% respectively Fig. 1.

**Fig. 2:**
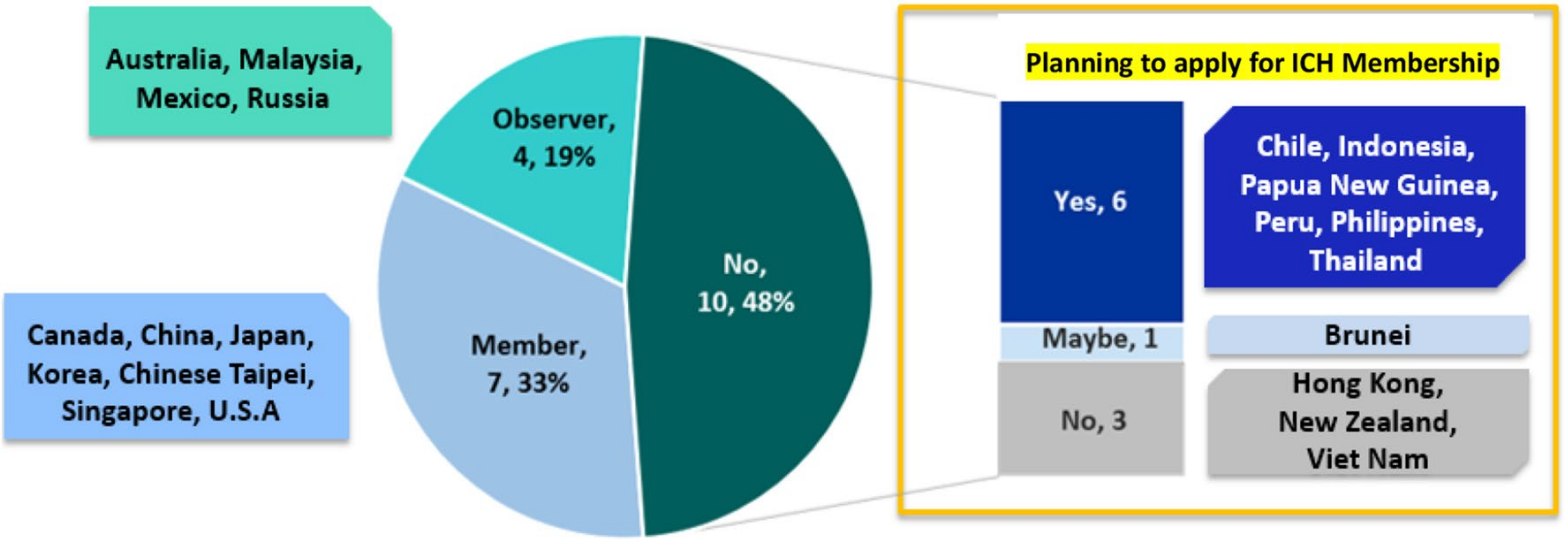
Full analysis of APEC economies ICH membership status in 2019

As of 2020, 33% of APEC RAs (7 of 21) are ICH members, while 62% (13 of 21) are PIC/S members. In addition to the 7 APEC RAs that are ICH members, 4 APEC RAs are observers and planning to apply for ICH membership Fig. 2. Among the 10 APEC RAs that are not ICH members, 6 economies are planning to apply for observership and ultimately membership in the future.

**Fig. 3:**
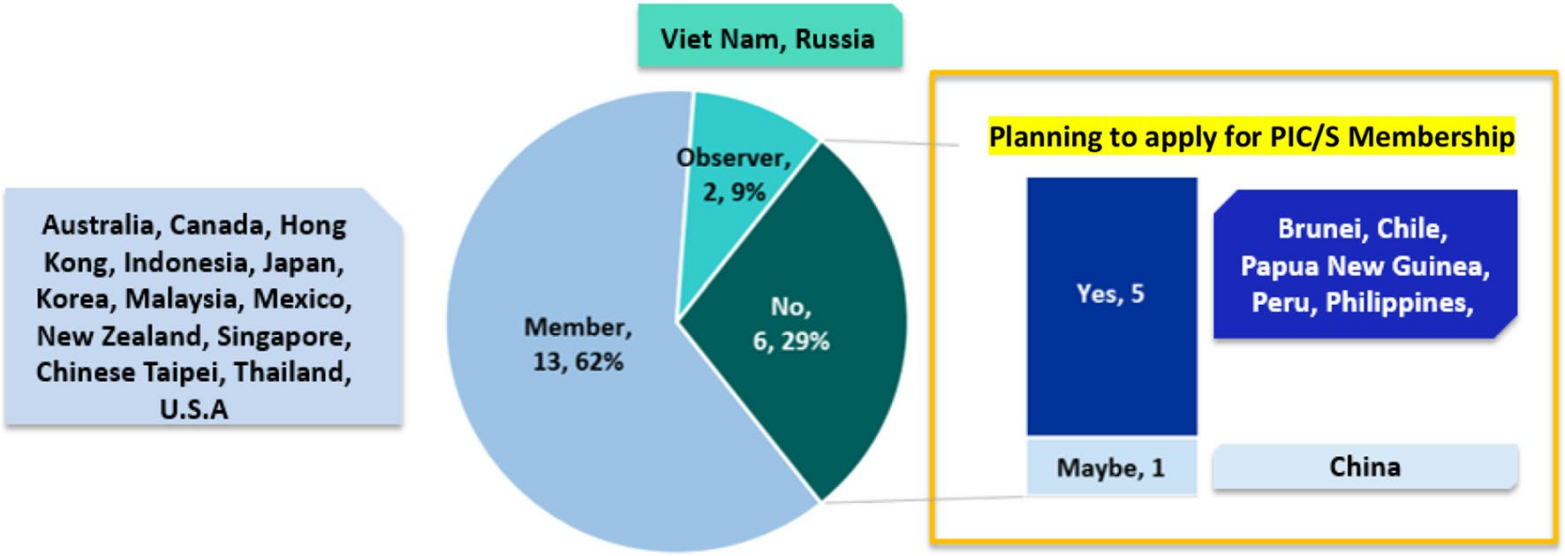
Full analysis of APEC economies PIC/S membership status in 2019

Figure 3 shows that among the 6 APEC RAs that are not PIC/S members, 100% are planning to apply for membership in the future.

### The Removal of CPP to Minimize Redundancy

Responses to Question: Do you require CPP issued by other RAs for registration application of drugs?

**Fig. 4:**
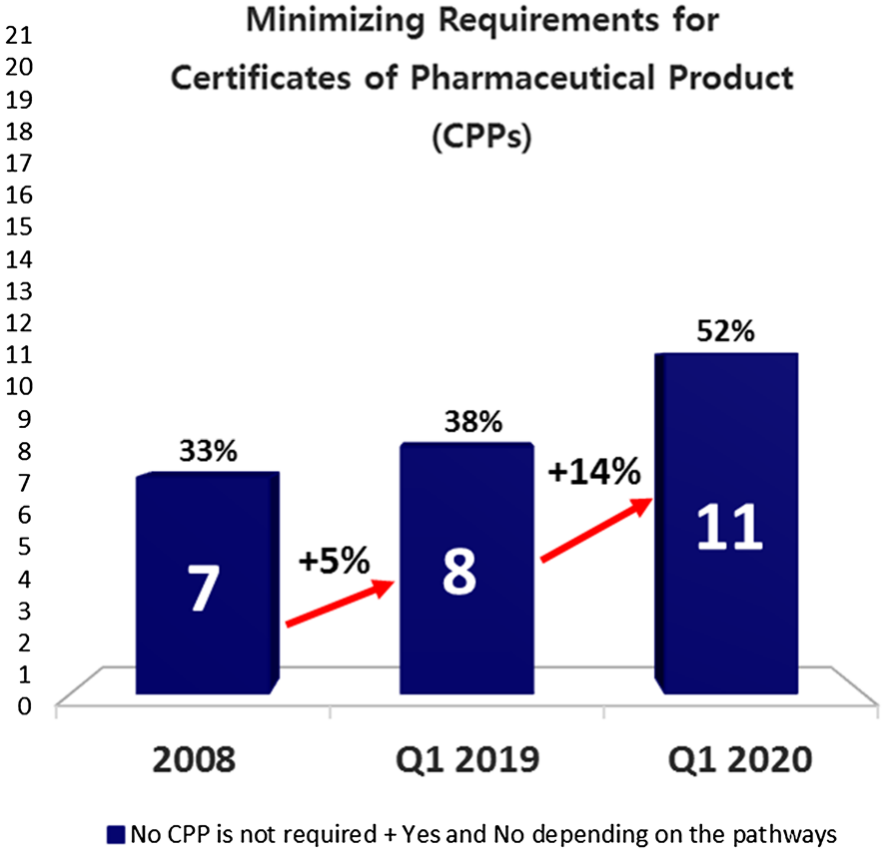
52% of APEC economies remove CPP since it is no longer used to replace full or partial review

In 2008, 7 economies including Australia, Canada, Mexico, Russia, Japan, Singapore and USA did not require a CPP from another reference agency for purposes of drug registration applications. Figure 4 shows that although only one additional economy minimized dependency on CPP by 2019 (5% increase over ten years), an additional three had adopted this approach by 2020 (14% increase over one year).^7^

In addition to Australia, Canada, Mexico, Russia, Japan, Singapore and USA, economies that removed CPP in 2020 are Papua New Guinea, China, Chinese Taipei and Chile. Among these economies, some of them may take a phased approach in removing CPP. As such, some economies may decide to remove CPP requirement for a particular pathway but not for all the pathways available in the local regulations. For example, in Chile, for initial marketing authorization (IMA) and line extension, CPP can be replaced by manufacturing agreement together with the approval letter (not mandatory but highly recommended) and at registration the application may be filed enclosing the GMP certificate and the contract that includes the product formula [11].

The increase in economies removing CPP could be attributed to the fact that as more economies mature and become members of ICH or PIC/S, they no longer need to depend on CPPs in their regulatory systems. This is in line with the observation that most of the APEC economies with ICH membership or observership shown in Fig. 2 do not require CPP for product evaluations.

The WHO introduced the CPP with the intention for recipient National Regulatory Authorities (NRAs) to rely on a prior trusted analysis to provide evidence of the quality of the products and replace full or partial regulatory assessments [31, 33]. However, the CPP process and use have not been revised since 1997 and countries have their own timing, terminology and format requirements. Moreover, contrary to its original intent of being a reliance tool for more harmonized process, the CPP has become an administrative tool.^8^ The exception to this and a good example of an economy that uses the CPP as a reliance tool is Hong Kong, where a CPP is used as evidence of robust assessment to reduce time to market [14].

In the event that the role of CPP, as defined by various APEC economies, differs from its original intent, then removing CPP use ensures that APEC economies will eliminate resource wastage in redundant document review activities that are part of their legacy processes. With the US FDA ceasing to issue CPP for products not exported out of the US from June 2020, APEC economies that depend on CPP should reconsider the role of CPP in their registration system [29]. If the use of CPP is mainly to confirm the approval status of a product in the country that issues CPP, then the approval letter or easily obtainable public assessment reports may be better suited for that purpose.

### The Use of PIC/S GMP Certificate to Minimize Duplication in Inspection

Responses to Question: Do you accept other RAs’ GMP certificates and to reduce the inspection burden?

**Fig. 5:**
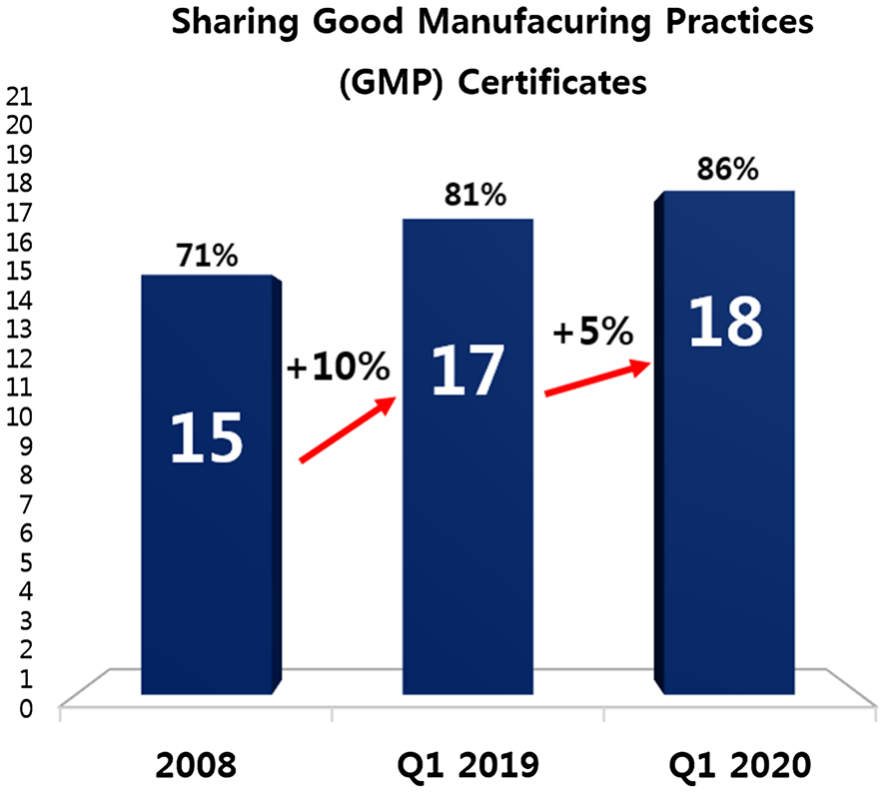
More than 85% of APEC economies accept other RAs’ GMP certificates to reduce inspection load

Compared to 15 APEC RAs that accepted other RAs’ GMP certificates in 2008, 17 (9.5% increase) and 18 (4.8% increase) optimally referenced PIC/S inspections by January 2019 and March 2020 respectively (Fig. 5).

**Fig. 6:**
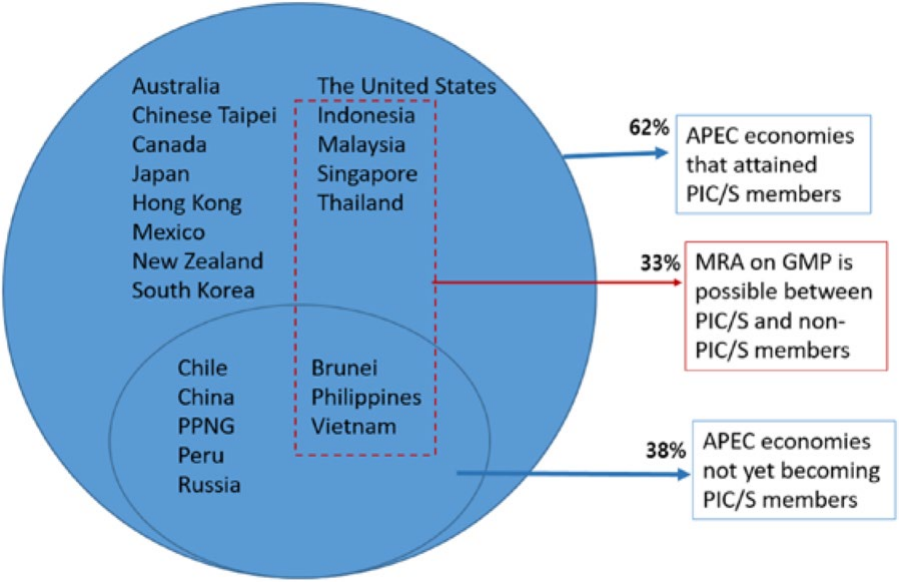
Reliance and mutual recognition arrangement exist among APEC economies to minimize duplication in inspection regardless of their PIC/S status

Increasing acceptance of GMP certificates issued by PIC/S members or under mutual recognition agreements (MRAs) to reduce portions of a full inspection requires practical collaboration, and this KPI is by far the best practice most used by APEC regulators to demonstrate convergence efforts. 18 APEC economies (> 85%) leverage on GMP certificates issued by PIC/S members, exceeding the 13 (62%) APEC economies attaining PIC/S status shown in Fig. 3. This demonstrates that APEC economies which are not yet PIC/S members also adopt this best practice to replace some, if not all, parts of a full inspection. In this regard, an excellent example of converging best practices to the highest degree is demonstrated by the MRA that exists among 7 APEC economies, namely Indonesia, Malaysia, Singapore, Thailand, Brunei, Philippines and Vietnam (Fig. 6).

This MRA is part of the economic integration of the Association of South East Asian Nations (ASEAN) and uses the PIC/S inspection framework whereby ASEAN Member States (AMS) agree to operate a PIC/S-equivalent GMP inspection framework. They are also obliged to accept GMP certificates or inspection reports issued by the listed Inspection Services, which are the AMS inspectorates whose GMP inspection systems meet the PIC/S framework [3, 28].

### The Management of Multiple Sites in Single License

Responses to Question: Do you manage multiple sites for the registration application of drugs?

Allowing a single license to cover multiple sites is a common practice of ICH members and often PIC/S members as well. The rise from 9 to 13 (19% increase) and subsequently to 15 (9% increase) APEC economies adopting this practice in 2019 and 2020 respectively compared to 2008 indicates progress in convergence.

**Fig. 7:**
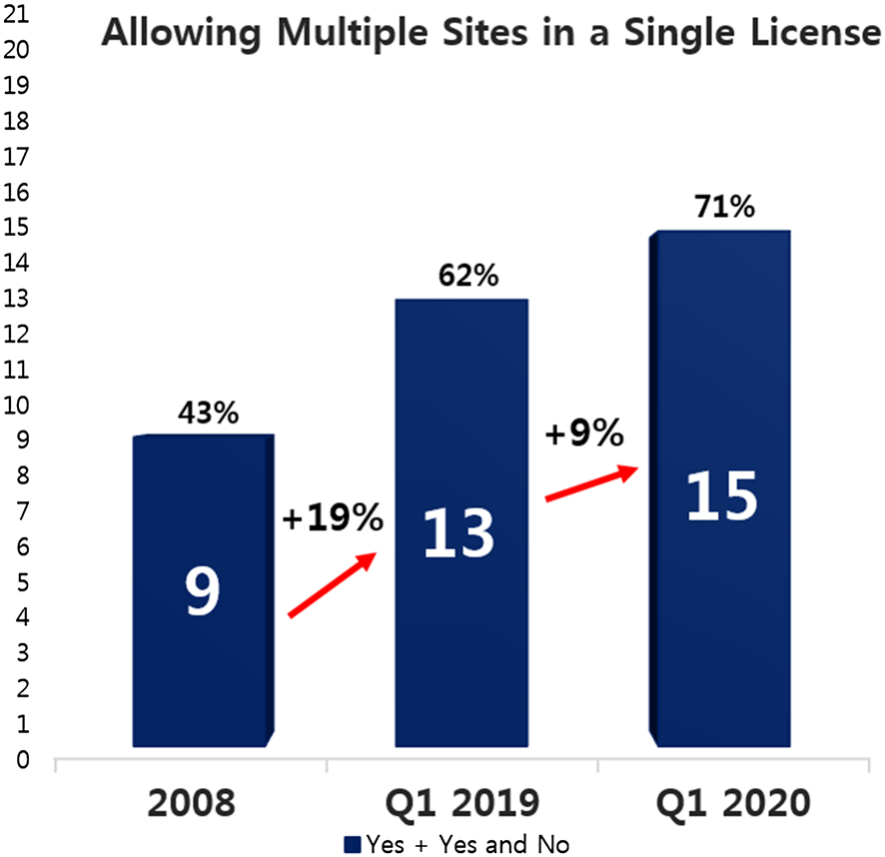
More than 70% of APEC economies practice multi-sites-in-a-license

The survey response received in Q1 2020 from the APEC economies indicated that 15 economies (Australia, Brunei, Canada, Japan, South Korea, New Zealand, Russia, Singapore, USA, China, Malaysia, Thailand, Mexico, Chinese Taipei and Indonesia) adopt the practice of multiple sites in a single license. Among them, some economies apply multiple sites in a single license for specific procedures only and not for all the evaluation pathways as shown in Fig. 7.

To increase supply, manufacturers need more sites to produce the drug products. The international standard of ICH and WHO recommends that an additional site be approved as a post-approval variation, and subsequently be included in the same license [17, 30]. The data required consists of relevant data generated from the new site, and is a reduced package compared to the full new drug application (NDA) package. In contrast, the one-site-one-license practice approves an additional site as a NDA with a new license issued at the end [4]. As such, a “repeated” full NDA package already approved in the IMA is mandatory in addition to the data generated from the new site. The new site must supply the full dataset as that required by an NDA including but not limited to a minimum of 12-months’ stability data, local testing and all country specific requirements. This approach complicates and hinders the addition of multiple sites, without enhancing the value of regulatory oversight. The recommendation is therefore for APEC economies to adopt the practice of multiple sites in a single license, in line with ICH and WHO guidances [17, 30].

### The Use of Risk-based Evaluation based on Reliance Practices

**Fig. 8:**
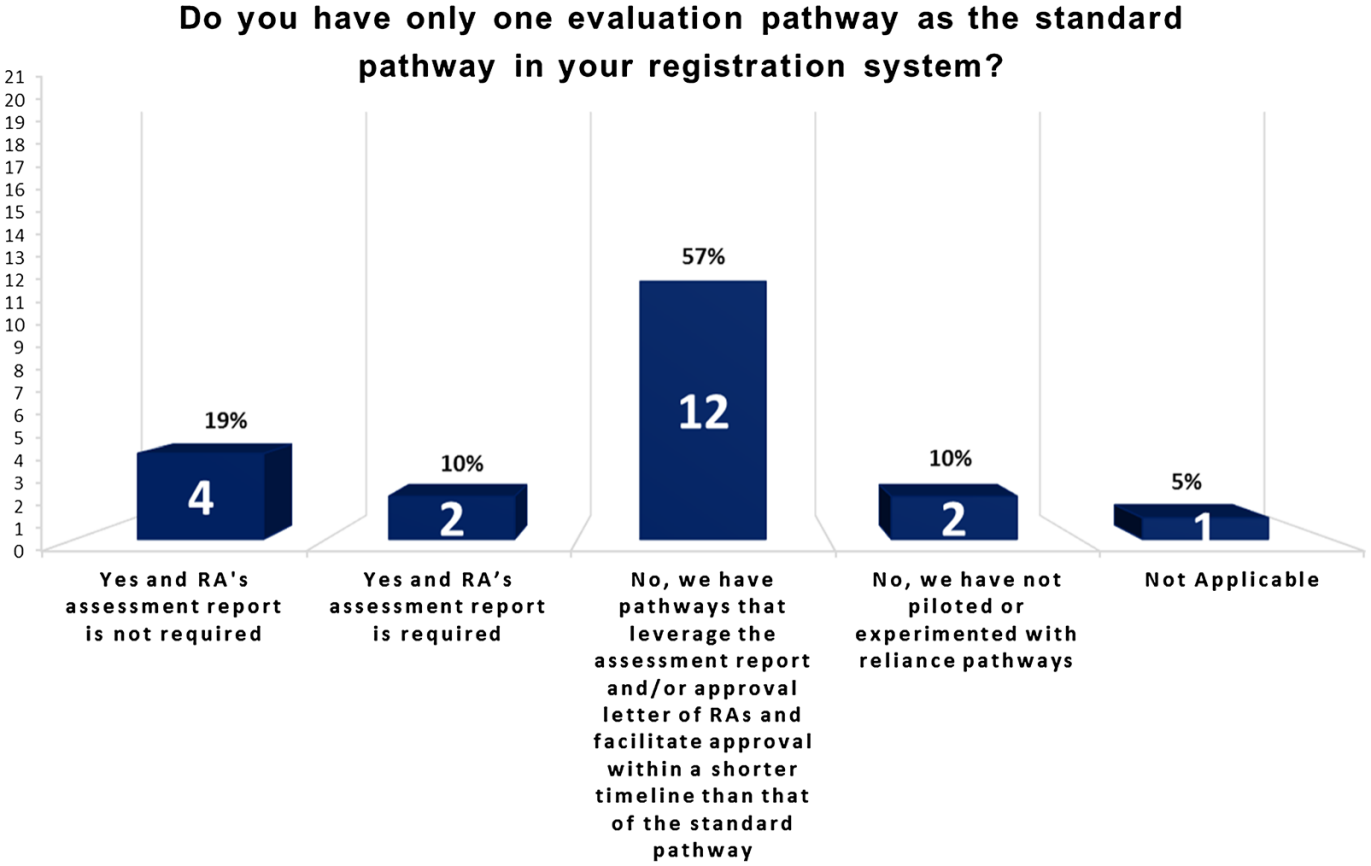
More than 50% of the APEC economies utilize reliance pathways

Figure 8 shows the status of APEC economies in practicing reliance in product approval as of March 2020. Twelve economies take into account and give significant weight to medical product assessments performed by other RAs in reaching their own decisions within a shorter timeline than that of their standard pathways. These 12 economies are Australia, Brunei, Hong Kong, Indonesia, Malaysia, Mexico, New Zealand, Peru, Papua New Guinea, Singapore, Chinese Taipei and Thailand. Previously in 2008, the additional pathway based on reliance practices existed in fewer APEC economies, which included Australia, Brunei, Hong Kong, New Zealand and Singapore. Although leveraging on the decisions of other RAs, the relying authorities remain independent, responsible and accountable regarding the decisions taken within their own jurisdictions [35]. The majority of the reliance pathways are applicable to NDA, although a few APEC economies, such as Singapore and Indonesia extend reliance pathways to cover post-approval variations as well [15, 21].

Joint reliance activity by two or more NRAs in order to work share and benefit from each other’s expertise also exists among the APEC economies. An example is the work sharing that Singapore subscribes to as part of the ACCESS consortium comprising of Australia, Canada, Singapore, Switzerland and the United Kingdom, to achieve product approval for marketing authorization [10]^9^. More recently, as part of Project ORBIS, the USA collaborated with regulators including regulators of the ACCESS consortium to conduct product evaluation in parallel and share the scientific rationale for decisions (Project Orbis). AMS that are part of APEC (Brunei, Indonesia, Malaysia, Philippines, Singapore, Thailand and Vietnam) have subscribed to a procedure for Strengthening the Implementation of Harmonized Regulatory Requirements (SIAHR) under the ASEAN Joint Assessment Coordinating Group (JACG) since 2017 [1]. This allows an applicant to submit the same product application simultaneously to two or more AMS to conduct their evaluations in parallel. The AMS participating in the ASEAN JACG may combine their lists of questions for the applicant and may also discuss or peer-review the evaluation reports, but each participating AMS eventually arrives at their respective regulatory decisions independently. See https://www.ifpma.org/resource-centre/regulatory-system-strengthening-for-health-products-in-asia-pacific/ for general overview.

**Fig. 9:**
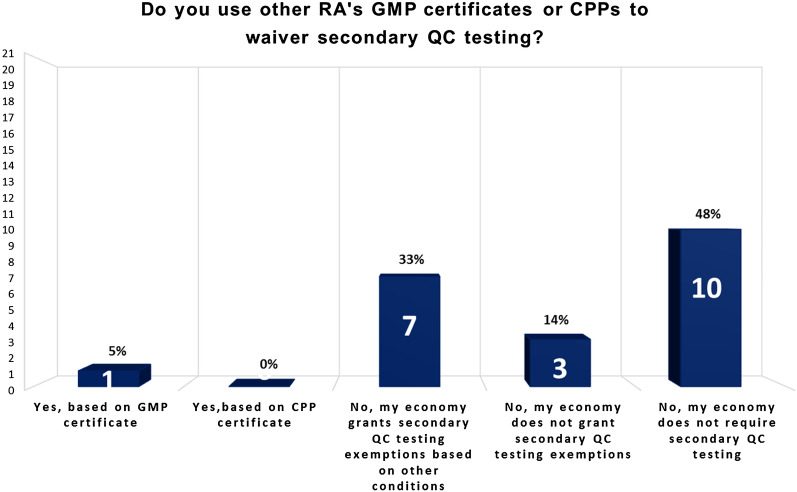
52% (11 economies) of APEC economies require secondary testing, out of which 1 economy can grant testing exemption based on reliance practices

Figure 9 presents the status of the APEC economies in practicing reliance to waive secondary quality control (QC) testing as of March 2020. In the scope of the survey, secondary QC testing includes testing required for importation or registration purposes. Currently ten of the APEC economies (48%) do not require secondary QC testing. These 10 economies are Australia, Brunei, Hong Kong, Indonesia, New Zealand, Philippines, Singapore, Chinese Taipei, Thailand and USA. Nearly 52% of the APEC economies continue to require secondary QC testing or import re-testing. Among these, 1 economy can rely on GMP certificates or CPP to waive import re-testing, while 7 economies may grant re-testing exemptions based on other conditions, such as technical or competence limitations (e.g. lack of equipment or method expertise) or low volume imports. However, 3 economies do not grant secondary QC testing exemptions. In contrast to the KPI result illustrated in Fig. 5 where 19 APEC economies leverage GMP certificates issued by PIC/S or MRAs countries to reduce inspection burden, the same 19 APEC economies may not apply the reliance approach in a similar way in waiving secondary testing.

## KPI Results Discussion and the Importance of Regulatory Convergence in the Context of the COVID-19 Pandemic

### APEC Member Economies Shifting Towards Regulatory Convergence

The survey results show the region shifting towards convergence. More APEC economies have attained full membership of ICH and PIC/S in the last decade. Joining regulatory convergence initiatives builds confidence and trust among regulators and enhances the necessary capacity to undertake regulatory convergence. With more regulatory authorities becoming members of ICH and PIC/S, regulatory requirements across the 21 economies are expected to become more aligned over time [9]. In addition, the number of economies removing CPP (as an administrative document) has risen from 7 in 2008 to 8 economies in 2019 over the decade, and then more significantly increasing by another 3 over just one year to 11 economies in 2020 (Fig. 4). Another gain includes the increase in the number of economies from 9 in 2008 to now over half (15 economies) of APEC economies agreeing to allow a single license to cover multiple sites. Removing the requirements for CPP as an administrative tool and allowing a single license to cover multiple sites are common practices that when widely adopted indicate convergence. Finally, the number of APEC economies recognizing GMP certificates issued by PIC/S increased from 15 in 2008 to 18 economies in 2020, which means far less duplication of inspection efforts. Acceptance of others’ GMP certificates requires real, practical collaboration. As such, increased acceptance is a hallmark of convergence [34]. As Dr. Nobumasa Nakashima of the Pharmaceuticals and Medical Devices Agency of Japan and co-chair of the RHSC has said, “When regulators take advantage of the testing, inspections and reviews already completed by other regulators, they save public resources while delivering quality medical products.” [7].

The use of reliance does not however correlate with all aspects; for example, 18 APEC economies accept GMP certificates to reduce inspection burden (Fig. 5) but only 1 economy accepts GMP certificates to waive secondary testing (Fig. 9). These figures suggest that the reliance approach is used inconsistently for inspections and secondary testing. A possible reason is that harmonization in the field of inspections was intensively driven by PIC/S whereas the updating of traditional testing requirements for purposes of reliance has yet to be realized. The harmonization of best practices coupled with robust quality management systems (QMS) is already in place to replace the need for redundant testing [19]. These appropriate control strategies should be considered to replace import re-testing which is an outdated practice that offers limited benefits to patients and may in some instances even increase risk to patients [22, 23, 27].

### The Importance of Regulatory Convergence in the Context of the COVID-19 Pandemic

Regulatory convergence plays an even more crucial role in the current COVID-19 pandemic – both to accelerate availability of COVID-19 vaccines, treatments and diagnostics and to maintain the availability of the existing medical products not related to COVID-19 emergency [35]. APEC’s early alignment in best practices coupled with information sharing and collaboration among regulators and manufacturers, facilitates appropriate regulatory oversight of novel or repurposed COVID-19 medicines, as well as diagnostics and vaccines throughout the products’ lifecycle management. As new COVID-19 therapies become available, all APEC economies are well positioned to avoid duplication and delays by quickly making full use of inspections conducted by other PIC/S members [36]. In addition, it is highly unlikely that onsite inspections can be conducted during the pandemic when inspectors are working remotely and traveling is severely restricted. As manufacturers need to increase capacity to supply COVID-19 medical products globally, convergence in areas, such as a single license for multi-sites facilitates dual sourcing so approvals of additional manufacturing sites can proceed quickly and supply with flexibility. Increased reliance ensures that rapid scientific assessment pathways for COVID-19 treatments and vaccines are in place. APEC economies can thus significantly leverage the assessment activities and reports of other NRAs to reach their own jurisdictional decisions speedily and without investing duplicative efforts. An example of reliance is use of the CPP by Hong Kong, China, where a CPP is used as evidence of robust assessment to reduce time to market [14]. In addition, several countries have leveraged reliance practices to eliminate redundancies and implement flexible approaches to secondary testing in view of the COVID-19 pandemic to ensure uninterrupted supply of medicines [20].

### The Role of Regulatory Cooperation and Reliance in the Context of the COVID-19 Pandemic

**Fig. 10:**
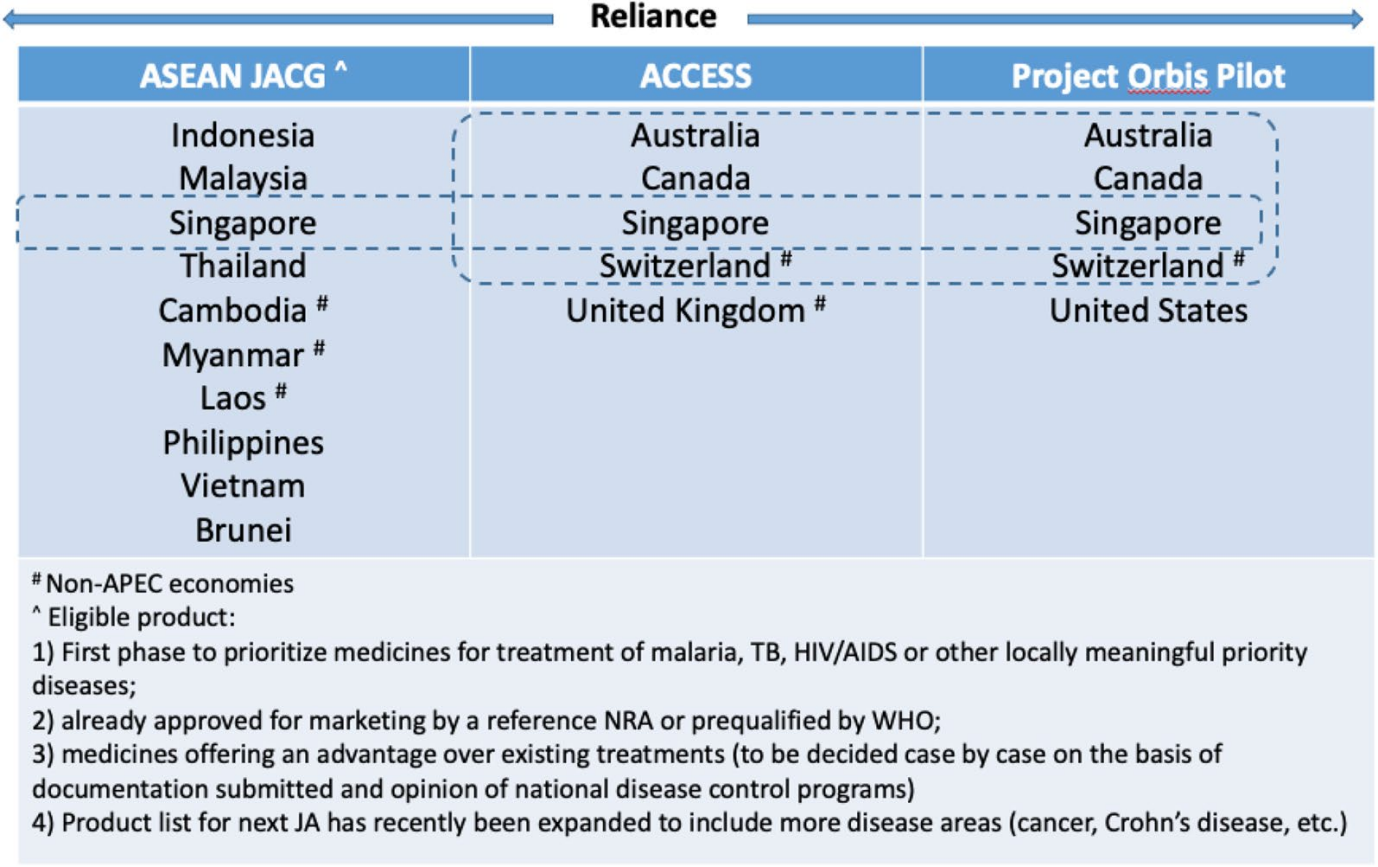
Reliance models that exist among the APEC economies

As the pandemic is global, regulatory cooperation across borders is paramount. An appropriately coordinated scientific assessment could facilitate an aligned outcome, enhancing public trust in the decision reached. Several APEC economies have already formed coordinated processes for assessing new drugs, such as ACCESS, ORBIS and the ASEAN Joint Assessment Coordinating Group (JACG) (Fig. 10). Although ORBIS is operational, and ACCESS continues to build on its proven ability to benefit from work sharing and commitment to review and collaborate on possible COVID-19 treatment options, the ASEAN JACG is still in its first phase with one product approved and a second under consideration [1, 10, 26]. There is an important opportunity to mobilize the ASEAN JACG for AMS to approve new products jointly, especially during these unprecedented times.

Reliance can also be a major accelerator to getting high-quality, safe and effective COVID-19 medical products to the population. ASEAN regulators, for example, will greatly benefit from its regional reliance mechanism to reduce regulatory duplication. Since there already exists a joint assessment process between Malaysia and Singapore,^10^ these 2 AMS can potentially take the lead in reviewing COVID-19 therapies, tapping into the existing framework of the ASEAN JACG and enabling other ASEAN regulators to make regulatory decisions expeditiously while preserving national sovereignty for issuing of regulatory approvals [2].

Given the powerful role reliance can play in accelerating the availability of vaccines, treatments and diagnostics, APEC member economies might consider pursuing additional reliance mechanisms. For example, with regard to the existing reliance models of ORBIS, ACCESS and the ASEAN JACG, there is room for enhancing the efforts in a way that these mechanisms can be leveraged collectively across multiple NRAs to bring optimal benefit to patients and populations in APEC and beyond. In particular, there is opportunity to initiate a joint assessment earlier or expedite it with good coordination to coincide with reliance on the decision(s) established by ACCESS or ORBIS. As a result, multiple NRAs in Fig. 10 would be able to arrive at their regulatory decisions about the same time. This would accelerate patient access to life saving medical products across more of the APEC region through joint, scientifically robust reviews. Such a collective mechanism would also enhance the capability of reviewers through work sharing and enable NRAs to utilize internal resources for further development of their regulatory systems [32]. APEC may consider whether it is feasible to pilot such a collective mechanism. This could be the springboard for larger scale collaboration in the future. The COVID-19 pandemic presents a pressing opportunity to significantly step-up convergence initiatives and related KPIs in order to rapidly address the current crisis and prepare for future public health emergencies [18].

Reliance practices recognize prior approvals by reference NRAs and enable patients around the globe to get timely access to not only COVID-19 related medical products but *all* medical products. With that, the recommendation is for NRAs to apply evaluation based on reliance practices to all disease areas, all product types, and all submission applications, including line and indication extensions and post-approval changes. NRAs can benefit from the APEC training in preparation for their implementation of reliance pathways. In the initial stage, NRAs are encouraged to consider implementing reliance with pilot projects which can be on a smaller scale, e.g. using a verification route for approving post-approval variations [35].

Within the APEC community, the economies can share their experience gained through ORBIS and ACCESS specifically on how the concept of reliance can address all regulatory functions spanning the full life cycle of medical products. This extends from clinical trial authorization to marketing authorization, including post-authorization procedures, vigilance, inspections, testing and lot release. Given the importance of post-approval changes to enable product supply on an unprecedented scale during public health emergencies, it is equally important to consider the use of reliance for pharmacovigilance and post-authorization activities to ensure regulatory oversight throughout the product life cycle. In addition, if an NRA has relied upon another NRA’s decision for its initial approval, there is a strong benefit for similar reliance measures for post-authorization changes and pharmacovigilance activities [35].

Although the decision to “regulate through reliance” is the hallmark of a modern and efficient regulatory authority, it is important to note that reliance does not equate to a loss of sovereignty. With regulatory reliance NRAs are taking into account and giving significant weight to—i.e. totally or partially relying upon – evaluations performed by another NRA or trusted institution in reaching its *own* decision. The relying authority remains responsible and accountable for decisions taken, even when it relies on the decisions and information of others [35].

## Conclusion

In 2009, APEC Ministers pledged to achieve the maximum level of convergence possible on approval procedures for medical products by 2020, to improve public access to life-saving innovations [9]. As Dr. Michelle Limoli of the U.S. Food and Drug Administration, who also serves as co-chair of the RHSC has stated, “Measurement leads to improvement. More regulatory convergence enhances the well-being of our people who rely on safe and effective medical products – and we look forward to achieving more progress.” [6]. The 2008–2020 survey conducted by the AHC shows marked progress in regulatory convergence. Regulatory requirements for the approvals of medical products are becoming more aligned for APEC economies. This is welcome progress that benefits people.

The outbreak of COVID-19 has laid bare the need to urgently improve regulatory cooperation to swiftly respond to public health emergencies and protect the world against future crisis [13]. It has also demonstrated the crucial role that regulators play to accelerate access to safe and effective medical products. The pandemic is a stark reminder that economies and livelihoods can be devastated without access to innovative medical treatments. Strong regulatory systems are a critical element of well-functioning health systems, a key contributor to improving access to high-quality and effective medical products, and ultimately to achieving universal health care [35]. Establishing and sustaining mature regulatory systems can be very resource intensive, requiring significant public investment. Therefore, NRAs must consider enhanced, innovative and more effective forms of cooperation in order to make the best use of the available resources and expertise and avoid duplication. This allows them to concentrate their regulatory efforts and resources where most needed [35]. APEC plays a key role in promoting and facilitating regulatory convergence, including reliance. NRAs can, through APEC’s network of Centers of Excellence for Regulatory Science, prepare to implement reliance pathways. Collectively, APEC may consider piloting a regional reliance program, whereby economies members co-create a functional mechanism that coordinated for multiple regulators to reach their regulatory decision around the same time.

As the world emerges from the immediate crisis, resources and priorities will need to be reviewed and reshaped. Although the KPIs discussed in this paper lend valuable insights for APEC member economies individually and collectively, new KPIs may be needed to enable a new era of innovation ushered in by COVID-19, such as those related to the adoption of telehealth and digital innovation [24]. Through a commitment to cooperation, training and innovation, the APEC RHSC can facilitate and accelerate access to life saving treatment solutions to benefit the health and wealth of all patients who reside in the region.

## Abbreviations

AHC: Asia Pacific Economic Cooperation (APEC) Harmonization Center
AMS: Association of South East Asian Nations (ASEAN) Member State
APEC: Asia Pacific Economic Cooperation
ASEAN: Association of South East Asian Nations
CPP: Certificate of Pharmaceutical Product
COE: Centres of Excellence
GMP: Good Manufacturing Practice
HSA: Health Sciences Authority
ICH: International Council for Harmonisation of Technical Requirements for Pharmaceuticals for Human Use
IMA: Initial Marketing Authorization
JACG: Joint Assessment Coordinating Group
KPI: Key Performance Indicator
LSIF: Life Sciences Innovation Forum
MRA: Mutual Recognition Arrangement
NDA: New Drug Application
PIC: Pharmaceutical Inspection Convention
PIC/S: The Pharmaceutical Inspection Co-operation Scheme
RHSC: Regulatory Harmonization Steering Committee
SIAHR: Strengthening the Implementation of Harmonized Regulatory Requirements
SRA: Stringent Regulatory Authorities
TGA: Therapeutic Goods Administration
USA: United States of America
USFDA: U.S. Food and Drug Administration

## Footnotes

1 Australia; Brunei Darussalam; Canada; Chile; People’s Republic of China; Hong Kong, China; Indonesia; Japan; Republic of Korea; Malaysia; Mexico; New Zealand; Papua New Guinea; Peru; The Philippines; The Russian Federation; Singapore; Chinese Taipei; Thailand; United States of America; Vietnam. https://www.apec.org/About-Us/About-APEC/Member-Economies

2 The APEC Harmonization Center is established under the authority of the APEC Life Sciences Innovation Forum (LSIF). Korea presented a proposal for a harmonization center, referred to as the AHC, at the sixth LSIF in August, 2008. https://www.nifds.go.kr/apec/content/view.do?contentKey=6&menuKey=42

3 Survey on Key Performance Indicator for APEC Regulatory Convergence https://www.nifds.go.kr/apec/article/view.do?articleKey=5514&searchTitleFlag=1&boardKey=132&menuKey=352&currentPageNo=1

4 Chong et al. “Developing Key Performance Indicators to measure the progress of regional convergence and cooperation in Asia Pacific Economic Cooperations (APEC)” AAPS Open [12] 4:4 https://doi.org/10.1186/s41120-018-0024-2

5 The International Council for Harmonisation of Technical Requirements for Pharmaceuticals for Human Use established in 1990 is an initiative that brings together regulatory authorities and the pharmaceutical industry to discuss scientific and technical aspects of pharmaceutical product development and registration.

6 The Pharmaceutical Inspection Co-operation Scheme established in 1995 is a non-binding, informal co-operative arrangement between regulatory authorities in the field of good manufacturing practice of medical products and comprises 54 participating authorities from around the world.

7 Rodier, C., Bujar, M., McAuslane, N. et al. Use of the Certificate for Pharmaceutical Products (CPP) in 18 Maturing Pharmaceutical Markets: Comparing Agency Guidelines with Company Practice. *Ther Innov Regul Sci* **55,** 118–128 (2021). https://doi.org/10.1007/s43441-020-00196-2https://link.springer.com/article/10.1007/s43441-020-00196-2

8 *ibid* Footnote 7, pp 119, 122.

9 https://www.hsa.gov.sg/therapeutic-products/international-collaboration/access

10 https://www.hsa.gov.sg/therapeutic-products/international-collaboration/npra
